# Association of Serum 25-Hydroxyvitamin D, Inflammatory, and Metabolic Biomarkers with Non-Proliferative Diabetic Retinopathy in Type 2 Diabetes Mellitus: A Cross-Sectional Study

**DOI:** 10.3390/biomedicines14081791

**Published:** 2026-08-09

**Authors:** Ana Maria Dascalu, Catalin Cicerone Grigorescu, Adriana Georgescu, Tudor Mihai Badescu, Cristina Alexandrescu, Daniela Stana, Madalina Totir, Anca Bobirca, Laura Carina Tribus, Marina Ionela Nedea, Crenguta Sorina Serboiu, Dragos Serban, Paul Lorin Stoica, Bogdan Mihai Cristea

**Affiliations:** 1 Doctoral School, University and Medicine “Carol Davila”, 020021 Bucharest, Romania; ana.dascalu@umfcd.ro (A.M.D.); dragos.serban@umfcd.ro (D.S.); 2 Faculty of Medicine, University and Medicine “Carol Davila”, 020021 Bucharest, Romaniaanca.bobirca@umfcd.ro (A.B.);; 3 Ophthalmology Department, Emergency University Hospital Bucharest, 050098 Bucharest, Romania; 4 Faculty of Medicine, “Vasile Goldis” Western University of Arad, 310025 Arad, Romania; 5 Faculty of Medicine, “Lucian Blaga” University of Sibiu, 550169 Sibiu, Romania; 6 Faculty of Dental Medicine, “Carol Davila” University of Medicine and Pharmacy Bucharest, 020021 Bucharest, Romania; laura.tribus@umfcd.ro; 7 Faculty of Pharmacy, University and Medicine “Carol Davila”, 020021 Bucharest, Romania; marina.nedea@umfcd.ro; 8 Fourth Department of General Surgery, Emergency University Hospital Bucharest, 050098 Bucharest, Romania

**Keywords:** type 2 diabetes mellitus (T2DM), non-proliferative diabetic retinopathy (NPDR), vitamin D, interleukin-6 (IL-6), neutrophil-to-lymphocyte ratio (NLR), inflammation, LDL-C/HDL-C ratio, biomarkers

## Abstract

**Background:** Non-proliferative diabetic retinopathy (NPDR) is a common microvascular complication of type 2 diabetes mellitus (T2DM) associated with chronic inflammation, metabolic dysregulation, and multiple interacting systemic factors. This study evaluated the associations of inflammatory biomarkers, serum 25-hydroxyvitamin D [25(OH)D], electrolyte homeostasis, and lipid-derived indices with NPDR. **Methods:** In this cross-sectional study with prospective recruitment, 98 patients with T2DM were classified into NPDR (*n* = 55) and non-DR (*n* = 43) groups. Clinical characteristics, hematological inflammatory indices, serum interleukin-6 (IL-6), C-reactive protein (CRP), 25(OH)D, electrolyte concentrations, lipid profile, and derived biomarkers—including the neutrophil-to-lymphocyte ratio (NLR), platelet-to-lymphocyte ratio (PLR), systemic immune–inflammation index (SII), LDL-C/HDL-C ratio, and calculated serum osmolarity—were compared. Receiver operating characteristic (ROC) analysis and multivariable logistic regression were performed to assess discriminatory performance and independent associations with NPDR. **Results:** Patients with NPDR had significantly lower serum 25(OH)D concentrations than the non-DR group (16.6 ± 7.5 vs. 21.0 ± 5.2 ng/mL, *p* < 0.001). Serum IL-6 levels (4.2 ± 3.1 vs. 2.8 ± 1.4 pg/mL, *p* = 0.047), NLR (*p* = 0.043), LDL-C/HDL-C ratio (*p* = 0.031), and calculated serum osmolarity (*p* = 0.033) were significantly higher, whereas lymphocyte count and the lymphocyte-to-monocyte ratio were significantly lower. Among individual biomarkers, serum 25(OH)D showed the best discriminatory performance (AUC = 0.712). A multivariable model including insulin therapy, IL-6, serum 25(OH)D, and the LDL-C/HDL-C ratio demonstrated good discrimination (AUC = 0.813), with 81.8% sensitivity and 81.4% specificity. Bootstrap validation supported the stability of the model. **Conclusions**: NPDR was associated with lower serum 25(OH)D concentrations, systemic inflammation, and a less favorable LDL-C/HDL-C ratio. A multivariable model integrating inflammatory, metabolic, and nutritional biomarkers showed better discriminatory performance than individual biomarkers. The association between insulin therapy and NPDR likely reflects greater diabetes severity rather than a direct effect of insulin. These findings are exploratory, and prospective studies in larger independent cohorts are needed to determine whether these biomarkers improve risk stratification beyond established clinical factors.

## 1. Introduction

In the context of global population aging and the increasing prevalence of obesity, sedentary lifestyles, and unhealthy dietary habits, type 2 diabetes mellitus (T2DM) has emerged as a major global public health challenge, imposing a substantial clinical and economic burden on healthcare systems worldwide. According to the International Diabetes Federation (IDF) Diabetes Atlas, 11th Edition, an estimated 589 million adults were living with diabetes in 2024, and this number is projected to rise to 853 million by 2050 [1]. Diabetic retinopathy (DR) is one of the most common microvascular complications of diabetes mellitus and remains a leading cause of preventable vision impairment and blindness among working-age adults worldwide [2,3]. Despite considerable advances in diabetes management and ophthalmic imaging, the increasing prevalence of T2DM continues to drive the global burden of diabetic retinopathy, emphasizing the need for improved strategies for early diagnosis, individualized risk stratification, and timely intervention [2].

Traditionally, DR has been attributed to chronic hyperglycemia-induced metabolic damage, including advanced glycation end-product accumulation, oxidative stress, polyol pathway activation, and progressive retinal microvascular injury [3,4,5]. However, recent evidence suggests that DR is a complex neurovascular inflammatory disease, in which retinal neurodegeneration, chronic low-grade inflammation, immune dysregulation, and microvascular dysfunction interact throughout disease development and progression [6,7,8,9]. Chronic systemic low-grade inflammation triggered by hyperglycemia and insulin resistance induces early impairment of the retinal neurovascular unit [8]. Persistent hyperglycemia activates retinal endothelial cells, Müller glial cells, microglia, and infiltrating leukocytes, leading to increased production of pro-inflammatory cytokines and angiogenic mediators, including interleukin-6 (IL-6), tumor necrosis factor-α (TNF-α), and vascular endothelial growth factor (VEGF) [8,9,10]. These mediators promote endothelial dysfunction, leukostasis, increased vascular permeability, breakdown of the blood–retinal barrier, capillary occlusion, retinal ischemia, and pathological neovascularization [7,8]. Among these inflammatory mediators, IL-6 has emerged as an important regulator linking systemic inflammation with retinal vascular injury and disease progression, both directly and indirectly through upregulation of VEGF expression [11,12]. Because IL-6 is readily measurable in peripheral blood and reflects both systemic inflammatory activity and retinal vascular dysfunction, it has attracted increasing interest as a potential biomarker for diabetic retinopathy risk stratification [12,13].

Because direct assessment of intraocular inflammation requires invasive sampling, increasing attention has focused on peripheral blood biomarkers as readily available surrogates of systemic inflammatory activity. Hematological indices derived from routine blood counts, including the neutrophil-to-lymphocyte ratio (NLR), platelet-to-lymphocyte ratio (PLR), and systemic immune–inflammation index (SII), have emerged as promising non-invasive markers reflecting the balance between innate immune activation and adaptive immune suppression [3,14,15]. In addition, circulating cytokines such as IL-6 and acute-phase reactants like C-reactive protein (CRP) may provide further insight into systemic inflammatory burden in diabetic patients [16,17]. However, their clinical utility for individualized risk stratification and prediction of diabetic retinopathy remains incompletely established.

Experimental studies indicate that vitamin D suppresses inflammatory cytokine production, modulates VEGF expression, improves endothelial function, and attenuates oxidative stress, mechanisms that may protect against retinal vascular injury [18,19]. Although several observational studies and recent meta-analyses have reported an inverse association between serum 25-hydroxyvitamin D concentrations and diabetic retinopathy, the independent contribution of vitamin D deficiency to disease progression remains incompletely understood [19,20,21].

Similarly, dyslipidemia contributes to endothelial dysfunction, oxidative stress, and chronic vascular inflammation. Recent evidence suggests that composite lipid-derived indices, particularly the LDL-C/HDL-C ratio and other atherogenic indices, may better reflect cardiovascular and microvascular risk than individual lipid parameters [22,23]. In addition, disturbances in electrolyte homeostasis—including alterations in calcium–magnesium balance and increased serum osmolarity—have been implicated in insulin resistance, endothelial dysfunction, and microvascular injury, although their role in diabetic retinopathy remains insufficiently investigated [24,25,26,27,28].

Although numerous studies have investigated individual inflammatory or metabolic biomarkers associated with diabetic retinopathy, relatively few have evaluated these biomarkers simultaneously within the same well-characterized cohort. Moreover, the independent predictive value of these biomarkers beyond established clinical risk factors such as glycemic control and diabetes duration remains insufficiently clarified.

Therefore, the aim of this study was to evaluate a comprehensive panel of systemic inflammatory, metabolic, and nutritional biomarkers—including IL-6, CRP, hematological inflammatory indices (NLR, PLR, SII), lipid-derived ratios, and vitamin D status—in patients with type 2 diabetes mellitus with and without diabetic retinopathy. We further sought to determine their independent association with diabetic retinopathy and evaluate their potential utility for risk stratification using multivariable logistic regression and receiver operating characteristic (ROC) analysis.

## 2. Materials and Methods

### 2.1. Study Design and Participants

We conducted a cross-sectional study with prospective recruitment, including T2DM patients admitted to the Department of Ophthalmology at the Emergency University Hospital Bucharest between March 2024 and March 2026. The study was designed to investigate the association between serum 25(OH)D, inflammatory and metabolic biomarkers, and the presence of non-proliferative diabetic retinopathy (NPDR). The study protocol was reviewed and approved by the Institutional Ethics Committee of the Emergency University Hospital Bucharest (Approval No. 7560/05.02.2024), and all procedures were conducted in accordance with the ethical principles of the Declaration of Helsinki. Written informed consent was obtained from all participants before enrollment, after a reasonable disclosure [29]. The manuscript was prepared in accordance with the Strengthening Reporting of Observational Studies in Epidemiology (STROBE) guidelines for observational studies.

### 2.2. Inclusion and Exclusion Criteria

Eligible participants were adults (≥18 years) with a confirmed diagnosis of T2DM based on established clinical criteria and with sufficient ocular media clarity to permit high-quality retinal imaging and ophthalmological examination.

Patients were excluded if they had conditions that could influence systemic inflammatory or metabolic parameters or confound ocular findings. Specifically, exclusion criteria included active infection, autoimmune or chronic inflammatory diseases, any type of malignancy, severe hepatic impairment, chronic kidney disease, recent use (within the previous three months) of corticosteroids or immunosuppressive agents, or iron or vitamin D supplementation during the same period. Patients with coexisting retinal diseases, including retinal vein occlusion, age-related macular degeneration, or pathological myopia, were also excluded to avoid confounding retinal pathology. To reduce heterogeneity, patients with proliferative diabetic retinopathy were excluded because proliferative disease is associated with substantially different inflammatory and immune profiles compared with NPDR. Admission for cataract surgery was not an exclusion criterion if retinal imaging and fundus examination could be performed.

**Figure 1:**
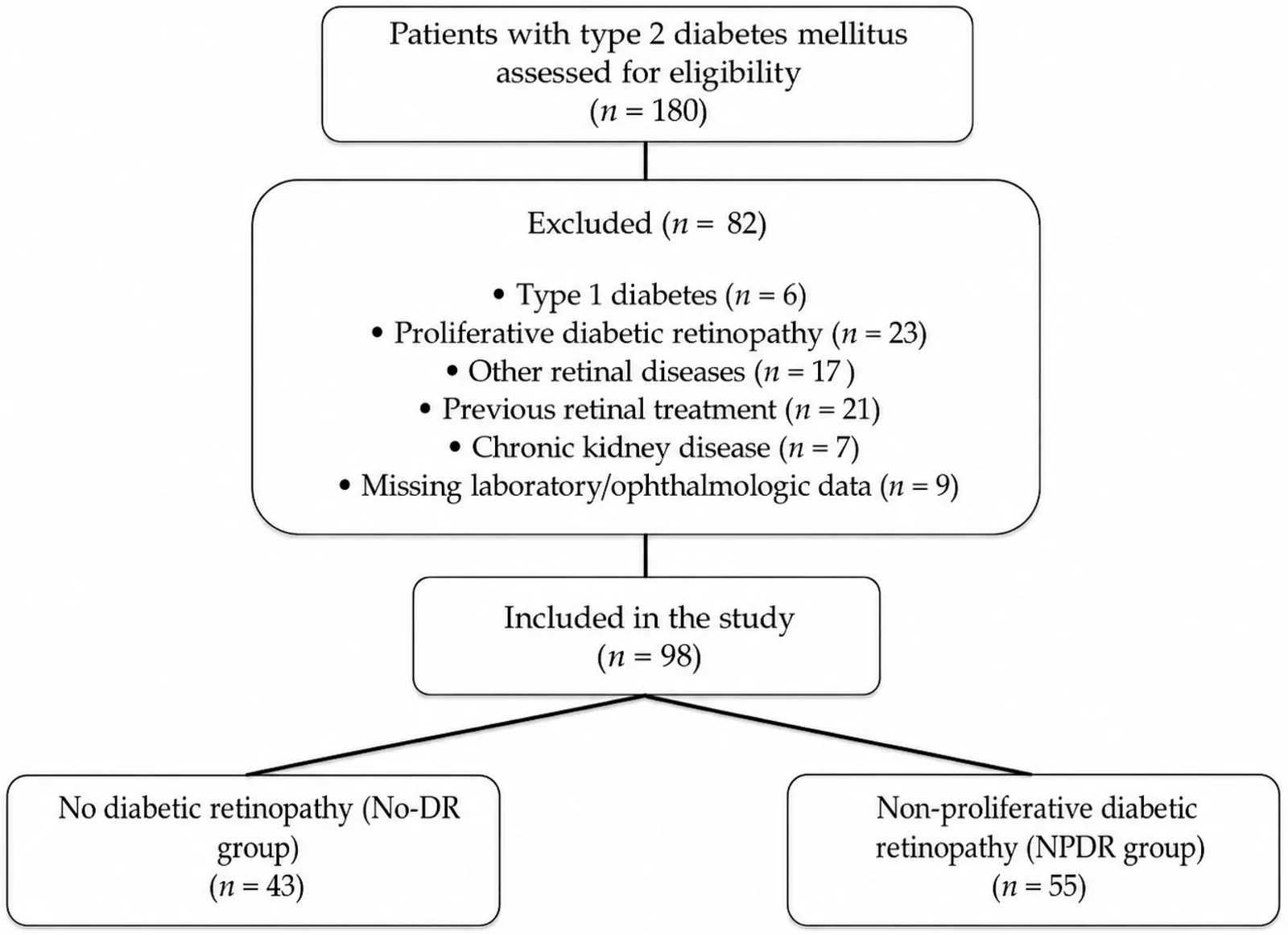
STROBE flow diagram of participant selection and study inclusion.

A total of 180 consecutive patients with type 2 diabetes mellitus admitted for evaluation to the Department of Ophthalmology between March 2024 and March 2026 were screened for eligibility. Patients were subsequently assessed according to the predefined eligibility criteria, and the participant selection process according to the STROBE recommendations is summarized in Figure 1.

Following the application of the eligibility criteria, 98 patients were included in the final analysis. Participants were stratified into two groups according to the presence of diabetic retinopathy: the non-DR group (*n* = 43), comprising patients without clinical or imaging evidence of diabetic retinopathy, and the NPDR group (*n* = 55), including patients with mild, moderate, or severe non-proliferative diabetic retinopathy according to the International Clinical Diabetic Retinopathy Disease Severity (ICDRS) Scale. Group allocation was based on a comprehensive ophthalmological examination supported by multimodal retinal imaging. Blood samples and ophthalmological examinations were performed on the same day prior to cataract surgery.

### 2.3. Ophthalmological Examination

All participants underwent a standardized comprehensive ophthalmological examination performed independently by two experienced retinal specialists. The examination included assessment of best-corrected visual acuity (BCVA) using a Snellen chart under standardized lighting conditions, measurement of intraocular pressure using Goldmann applanation tonometry, slit-lamp biomicroscopy of the anterior segment, and dilated fundus examination using a 90-diopter lens.

The diagnosis and staging of diabetic retinopathy were confirmed using color fundus photography (Topcon non-mydriatic retinal camera, Tokyo, Japan). In addition, spectral-domain optical coherence tomography (CIRRUS 5000 OCT system, Carl Zeiss Meditec, Oberkochen, Germany) was performed in all participants using the Macular Cube 512 × 128 acquisition protocol to evaluate retinal structural changes, including central macular thickness and the presence of diabetic macular edema. Diabetic retinopathy severity was graded according to the International Clinical Diabetic Retinopathy Disease Severity Scale as no diabetic retinopathy, mild NPDR, moderate NPDR, or severe NPDR. For patients with bilateral diabetic retinopathy, the eye with the more advanced retinopathy grade was used for patient-level classification. Thus, patients were classified according to the more severely affected eye, while patients without retinopathy in either eye were classified as non-DR. This approach was selected to capture the maximum clinically documented degree of retinal involvement in patients with asymmetric bilateral disease. Any discrepancies between the two examiners were resolved by consensus.

### 2.4. Laboratory Measurements

Venous blood samples were collected from all participants in the morning after an overnight fast of at least 8 h to minimize metabolic variability.

Complete blood count (CBC) parameters, including total leukocyte, neutrophil, lymphocyte, monocyte, platelet, and red blood cell counts, hemoglobin (Hb), and red cell distribution width (RDW), were analyzed using an automated hematology analyzer (DxH 800 Hematology Analyzer, Beckman Coulter, Brea, CA, USA) according to the manufacturer’s standardized protocols.

Biochemical analyses were performed using standard automated enzymatic and colorimetric methods and included fasting blood glucose (FBG), glycated hemoglobin (HbA1c), blood urea nitrogen (BUN), serum creatinine, uric acid, liver transaminases, lipid profile (total cholesterol, high-density lipoprotein cholesterol [HDL-C], low-density lipoprotein cholesterol [LDL-C], and triglycerides), electrolytes (calcium, magnesium, sodium, and potassium), C-reactive protein (CRP), and serum iron.

Serum 25(OH)D and interleukin-6 (IL-6) concentrations were quantified using a UniCel DxI 800 analyzer (Beckman Coulter, Brea, CA, USA) based on a solid-phase chemiluminescent enzyme immunoassay. According to the 2023 updated clinical practice guidelines, vitamin D status was classified as deficient (<20 ng/mL), insufficient (20–29 ng/mL), and sufficient (≥30 ng/mL) [20,30]. Severe vitamin D deficiency was defined as a serum 25(OH)D concentration below 12 ng/mL [31].

### 2.5. Calculated Biomarkers

To further characterize metabolic, inflammatory, and cardiovascular risk profiles, several derived biomarkers were calculated from routine laboratory parameters. The calcium-to-magnesium ratio (Ca/Mg), reflecting calcium–magnesium homeostasis, was calculated using serum calcium and magnesium concentrations expressed in mg/dL.

Serum osmolarity was estimated using the Smithline-Gardner formula [24]:

Serum osmolarity (mOsm/L) = (2 × serum sodium [mmol/L]) + (glucose [mg/dL]/18) + (BUN [mg/dL]/2.8)(1)

Atherogenic lipid indices were calculated to assess cardiovascular risk. The Castelli Risk Index I (CRI-I) was calculated as the ratio of total cholesterol to HDL-C. The LDL-C/HDL-C ratio was calculated by dividing LDL-C by HDL-C. The atherogenic index of plasma (AIP) was calculated using triglyceride and HDL-C concentrations expressed in mmol/L:

AIP = log10 [triglycerides (mmol/L)/HDL-C (mmol/L)](2)

The hemoglobin-to-red cell distribution width ratio (HRR), a marker of hematological and inflammatory status, was calculated as

HRR = hemoglobin (g/dL)/RDW (%)(3)

Inflammatory indices derived from complete blood count parameters included the neutrophil-to-lymphocyte ratio (NLR), calculated as the ratio of the absolute neutrophil count to the absolute lymphocyte count; the platelet-to-lymphocyte ratio (PLR), calculated as the ratio of the platelet count to the absolute lymphocyte count; and the systemic immune–inflammation index (SII), calculated as

SII = [neutrophils (×10^3^/µL) × platelets (×10^3^/µL)]/lymphocytes (×10^3^/µL) (4)

Neutrophil, lymphocyte, and platelet counts were expressed as absolute values (×10^3^/µL).

### 2.6. Statistical Analysis

Statistical analyses were performed using EasyMedStat software (version 4.5; EasyMedStat, Levallois-Perret, France) and MedCalc Statistical Software (version 23.1.7; MedCalc Software Ltd., Ostend, Belgium).

Continuous variables are presented as mean ± standard deviation (SD) or median with interquartile range (IQR), depending on data distribution. Categorical variables are presented as frequencies and percentages. Normality was assessed using the Shapiro–Wilk test. Homogeneity of variances was assessed using Levene’s test. Between-group comparisons were performed using the independent-samples Student’s *t*-test, Welch’s *t*-test, or the Mann–Whitney *U* test, as appropriate, according to data distribution and variance homogeneity. Categorical variables were compared using the χ^2^ test or Fisher’s exact test, as appropriate. Given the exploratory nature of the study and the number of biomarkers evaluated, the possibility of type I error due to multiple comparisons was considered. No formal multiplicity correction was applied to the primary univariate comparisons because the study was intended to generate hypotheses regarding potentially relevant inflammatory, nutritional, metabolic, and electrolyte-related biomarkers. Accordingly, statistically significant findings were interpreted in conjunction with effect estimates, confidence intervals, ROC analyses, and multivariable logistic regression rather than on the basis of *p*-values alone. The potential for false-positive findings was considered when interpreting the results.

Receiver operating characteristic (ROC) curve analysis was performed to evaluate the diagnostic performance of inflammatory biomarkers. The area under the curve (AUC), optimal cut-off values determined using the Youden index, sensitivity, specificity, and corresponding 95% confidence intervals were calculated.

To identify independent predictors of NPDR, multivariable logistic regression analysis was performed respecting the EPV (event-per-variable) rule. Odds ratios (ORs) with 95% confidence intervals (CIs) were reported. All statistical tests were two-sided, and a *p*-value < 0.05 was considered statistically significant.

Potential multicollinearity among predictors was assessed using Spearman correlation analysis and variance inflation factors (VIFs). VIF values were obtained from an auxiliary least-squares regression analysis of the predictor variables. VIF values < 5 were considered indicative of the absence of relevant multicollinearity.

### 2.7. Risk of Bias

Several measures were implemented to minimize potential bias. Consecutive patient recruitment was used to reduce selection bias. Ophthalmological examinations were independently performed by two experienced retinal specialists according to standardized protocols, and disagreements were resolved by consensus. Laboratory analyses were performed using standardized automated methods in the same hospital laboratory. Participants with missing laboratory or ophthalmological data were excluded from the final statistical analysis.

## 3. Results

### 3.1. Baseline Clinical and Demographic Characteristics

A total of 98 patients with type 2 diabetes mellitus (T2DM) were included in the final analysis, comprising 55 patients with non-proliferative diabetic retinopathy (NPDR) and 43 patients without diabetic retinopathy (non-DR). The mean age of the study population was 66.5 ± 8.5 years, with no significant difference between groups. Baseline demographic, clinical, and biochemical characteristics are summarized in Table 1.

Patients with NPDR were more frequently treated with insulin (61.8% vs. 16.2%, *p* < 0.001), whereas treatment with oral antidiabetic drugs alone was more common in the non-DR group (83.7% vs. 50.9%, *p* = 0.002). Serum uric acid levels were significantly lower in patients with NPDR than in those without DR (5.2 ± 1.6 vs. 5.9 ± 1.6 mg/dL, *p* = 0.031).

No significant differences were observed between groups regarding age, sex distribution, duration of diabetes, prevalence of arterial hypertension, fasting blood glucose, HbA1c, renal function parameters (blood urea nitrogen and serum creatinine), or liver enzyme levels (ALT and AST), although patients with NPDR showed a trend toward a longer duration of diabetes and higher fasting blood glucose and HbA1c levels. Renal function was additionally assessed by a retrospectively calculated eGFR using the 2021 CKD-EPI creatinine-based equation. Mean eGFR did not differ significantly between patients with NPDR and those without DR (*p* = 0.731). A reduced eGFR (<60 mL/min/1.73 m^2^) was more frequent among patients with NPDR than among those without DR (32.7% vs 18.6%), although this difference did not reach statistical significance (*p* = 0.116).

### 3.2. Hematologic and Inflammatory Indices

Patients with NPDR exhibited significant alterations in several hematological and inflammatory biomarkers compared with the non-DR group. Absolute lymphocyte counts were significantly lower in patients with NPDR than in those without diabetic retinopathy (2.1 ± 0.6 vs. 2.4 ± 1.0 × 10^3^/µL, *p* = 0.047). Consequently, the neutrophil-to-lymphocyte ratio (NLR) was significantly higher in the NPDR group (2.8 ± 1.2 vs. 2.3 ± 0.6, *p* = 0.043), whereas the lymphocyte-to-monocyte ratio (LMR) was significantly lower (3.2 ± 1.2 vs. 3.7 ± 1.4, *p* = 0.023) (Table 2).

Similarly, serum IL-6 concentrations were significantly elevated in patients with NPDR compared with those without diabetic retinopathy (*p* = 0.047), supporting the presence of a higher inflammatory burden in patients with retinal microvascular involvement.

Although SII, PLR, platelet count, fibrinogen concentration, erythrocyte sedimentation rate (ESR), and C-reactive protein (CRP) levels were numerically higher in the NPDR group, these differences did not reach statistical significance. Likewise, no significant between-group differences were observed in red blood cell count, hemoglobin concentration, red cell distribution width (RDW), and hemoglobin-to-RDW ratio (HRR).

Taken together, these findings suggest that NPDR is associated with subtle but measurable systemic inflammatory changes, primarily characterized by lymphocyte depletion, increased NLR, reduced LMR, and elevated circulating IL-6 concentrations.

### 3.3. Serum 25(OH)D, Electrolytes, and Osmolarity

Serum 25(OH)D levels were significantly lower in patients with DR compared with non-DR patients (16.7 ± 7.5 vs. 21.0 ± 5.2 ng/mL, *p* < 0.001). Vitamin D deficiency was significantly more prevalent in the NPDR group (76.4% vs. 44.2%, *p* = 0.002), as was severe vitamin D deficiency (29.1% vs. 2.3%, *p* < 0.001) (Table 3).

Serum calcium, magnesium, sodium, iron, total iron-binding capacity (TIBC), transferrin saturation (TSAT), and the calcium-to-magnesium (Ca/Mg) ratio did not differ significantly between the two groups (all *p* > 0.05). Although the mean Ca/Mg ratio exceeded 5 in both groups, no significant between-group difference was observed (*p* = 0.943). Serum potassium concentrations remained within the reference range (3.5–5.1 mmol/L) in both groups. However, patients with NPDR exhibited a marginally higher mean potassium concentration than those without DR (4.6 ± 0.6 vs. 4.4 ± 0.4 mmol/L), reaching borderline statistical significance (*p* = 0.050).

Estimated serum osmolarity was significantly higher in the NPDR group compared with the non-DR group (309.0 ± 14.2 vs. 303.4 ± 7.6 mmol/L, *p* = 0.033). This difference may reflect the combined contribution of metabolic and renal factors, as patients with NPDR also demonstrated higher fasting blood glucose (159.5 ± 70.7 vs. 128.4 ± 35.7 mg/dL) and blood urea nitrogen (54.7 ± 34.3 vs. 43.6 ± 15.9 mg/dL) values.

### 3.4. Lipid Profile and Atherogenic Indices

A comparative evaluation of the lipid profile showed that although total cholesterol, triglycerides, HDL-C, and LDL-C concentrations did not differ significantly between groups, the LDL-C/HDL-C ratio was significantly higher in patients with NPDR (2.3 ± 1.0 vs. 1.9 ± 0.9, *p* = 0.031), indicating a less favorable balance between atherogenic and antiatherogenic lipoproteins in patients with retinal involvement (Table 4).

Both groups exhibited relatively elevated AIP values (0.5 ± 0.3 in the NPDR group and 0.5 ± 0.2 in the non-DR group), with no significant difference between groups. These findings suggest an overall unfavorable atherogenic profile in the study population but do not support a specific association between AIP and NPDR.

### 3.5. Diagnostic Performance of Selected Systemic Metabolic and Inflammatory Biomarkers for Identifying NPDR in Patients with T2DM

**Figure 2:**
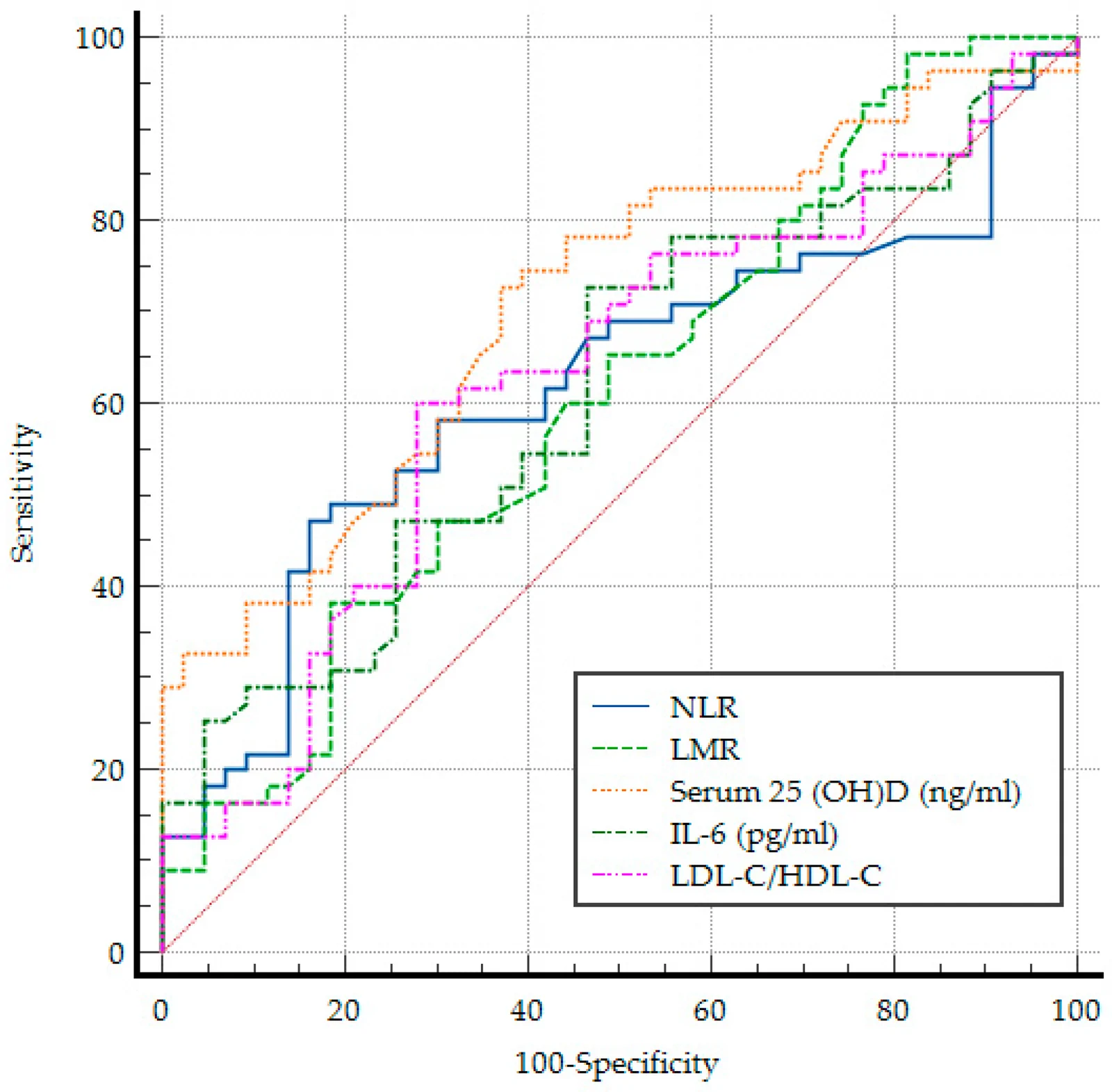
Comparative ROC curves for NLR, LMR, IL-6, 25(OH)D, and LDL-C/HDL-C ratio.

Receiver operating characteristic (ROC) curve analysis was performed to evaluate the ability of selected metabolic and inflammatory biomarkers to discriminate between patients with and without NPDR (Table 5, Figure 2).

Among the evaluated biomarkers, serum 25(OH)D demonstrated the best discriminatory performance (AUC = 0.712), with a sensitivity of 72.7% and a specificity of 62.8% at the optimal cut-off value. The remaining biomarkers, including NLR, LMR, IL-6, and LDL-C/HDL-C ratio, exhibited only poor-to-fair discriminative ability (AUCs < 0.65), indicating limited diagnostic performance when used individually.

An exploratory analysis was performed to assess whether the investigated biomarkers differed according to NPDR severity. Patients were categorized into non-DR (*n* = 43), mild NPDR (*n* = 9), moderate NPDR (*n* = 22), and advanced NPDR (*n* = 24). Using the Kruskal–Wallis test, serum 25(OH)D levels differed significantly across the four groups (*p* = 0.004), with lower levels observed in patients with moderate and advanced NPDR compared with those without DR on Dunn’s post hoc analysis. No statistically significant differences across severity categories were observed for IL-6 (*p* = 0.149), NLR (*p* = 0.053), PLR (*p* = 0.744), SII (*p* = 0.671), or LDL-C/HDL-C ratio (*p* = 0.074). Given the relatively small number of patients with mild NPDR (*n* = 9), these subgroup analyses were considered exploratory (Supplementary Material).

#### Multivariate Logistic Regression Analysis

In the multivariable logistic regression model, serum 25(OH)D, IL-6, LDL-C/HDL-C ratio, and the need for insulin therapy were independently associated with NPDR.

Spearman correlation analysis demonstrated moderate-to-strong correlations among several hematological inflammatory indices, particularly SII with PLR (ρ = 0.708, *p* < 0.0001), SII with NLR (ρ = 0.703, *p* < 0.0001), and NLR with LMR (ρ = −0.607, *p* < 0.0001). To avoid multicollinearity, these correlated inflammatory indices were not entered simultaneously into the final multivariable logistic regression model. Among the predictors included in the final model, pairwise correlations were weak, with the strongest correlation observed between serum 25(OH)D and insulin therapy (ρ = −0.297, *p* = 0.003) (Supplementary Material). Auxiliary VIF analysis showed values ranging from 1.040 to 1.129 for the predictors retained in the final model, indicating no relevant multicollinearity.

**Figure 3:**
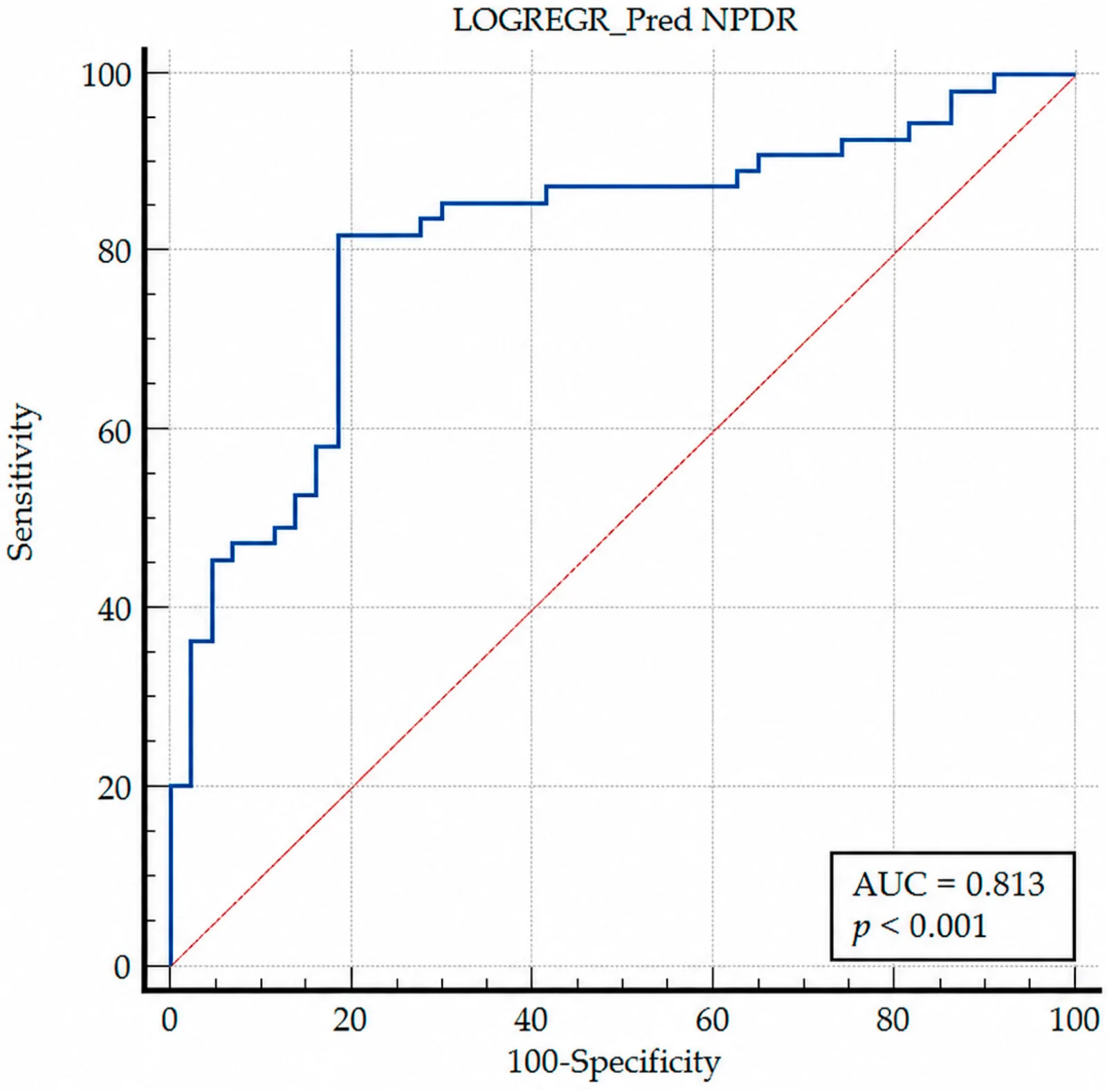
ROC curve for the multivariate model predicting NPDR in the study group.

Lower 25(OH)D levels were associated with higher odds of NPDR (OR = 0.918, 95% CI: 0.850–0.990, *p* = 0.027), whereas higher IL-6 concentrations (OR = 1.496, 95% CI: 1.097–2.039, *p* = 0.011) and a higher LDL-C/HDL-C ratio (OR = 2.017, 95% CI: 1.166–3.489, *p* = 0.012) were associated with increased odds of NPDR. Insulin therapy was significantly associated with NPDR in the multivariable model (OR = 4.516, 95% CI: 1.472–13.852, *p* = 0.008), although this association should be interpreted cautiously because insulin requirement may reflect greater diabetes severity and treatment intensity (Table 6, Figure 3).

This model demonstrated good discriminatory performance, with an AUC of 0.813, a sensitivity of 81.8%, and a specificity of 81.4%. The overall model was statistically significant (χ^2^ = 32.235, df = 4, *p* < 0.001), with a Nagelkerke R^2^ of 0.376. The Hosmer–Lemeshow test did not indicate significant evidence of poor model fit (*p* = 0.125). Internal validation was performed using 1000 bootstrap resamples to estimate optimism-corrected model performance. The bootstrap BCa 95% confidence interval for the AUC was 0.710–0.893, which was similar to the DeLong confidence interval (0.722–0.885), providing supportive evidence of the stability of the model’s discriminatory performance. External validation in an independent cohort was not performed and should be considered in future studies.

In a sensitivity analysis incorporating eGFR as a continuous covariate into the final multivariable logistic regression model, the associations of IL-6, 25(OH)D, LDL-C/HDL-C ratio, and insulin therapy with NPDR remained essentially unchanged, whereas eGFR was not independently associated with NPDR (OR = 1.003, 95% CI: 0.983–1.025, *p* = 0.747).

To assess whether the observed associations were robust to conventional measures of diabetes duration and glycemic status, two sensitivity analyses were performed. HbA1c and fasting blood glucose (FBG) were evaluated in separate models because they represent overlapping measures of glycemic status. In both sensitivity analyses, serum 25(OH)D, IL-6, and the LDL-C/HDL-C ratio remained independently associated with NPDR, whereas neither diabetes duration nor HbA1c (Model 1) or FBG (Model 2) showed independent associations (Table 7).

The persistence of these associations across both sensitivity analyses supports their robustness to adjustment for conventional measures of diabetes duration and glycemic status. These findings suggest that the association between insulin therapy and NPDR may capture aspects of diabetes severity or treatment dependence that are not fully represented by diabetes duration or single measures of glycemic control.

## 4. Discussion

This cross-sectional study evaluated the relationship between inflammatory, metabolic, and nutritional biomarkers and the presence of non-proliferative diabetic retinopathy (NPDR) in patients with type 2 diabetes mellitus (T2DM). Our findings indicate that NPDR is associated with a constellation of systemic abnormalities rather than a single biochemical disturbance. Insulin therapy showed a strong association with NPDR. This finding likely reflects greater disease severity, treatment dependence, longer diabetes duration, or poorer glycemic control among patients requiring insulin rather than a direct causal effect of insulin therapy on retinal disease. Patients with NPDR exhibited lower serum 25(OH)D concentrations, higher circulating IL-6 levels, increased neutrophil-to-lymphocyte ratio (NLR), lower lymphocyte-to-monocyte ratio (LMR), higher calculated serum osmolarity, and a less favorable LDL-C/HDL-C ratio. However, the discriminatory performance of individual biomarkers in univariate ROC analyses was generally poor to fair. In the multivariable model, insulin therapy, IL-6, the LDL-C/HDL-C ratio, and serum 25(OH)D concentrations remained independently associated with NPDR, suggesting that inflammatory activation, metabolic dysregulation, and nutritional status represent complementary components of diabetic retinal disease. Importantly, additional adjustment for eGFR did not materially alter these associations, and eGFR itself was not independently associated with NPDR, suggesting that the observed relationships were unlikely to be substantially confounded by renal function.

These findings are consistent with the current understanding of diabetic retinopathy as a chronic neurovascular disorder rather than merely a microvascular complication of hyperglycemia [7,8]. Increasing evidence indicates that retinal neurodegeneration and chronic low-grade inflammation occur early in the disease process and contribute to microvascular dysfunction, supporting the concept of diabetic retinopathy as a neurovascular inflammatory disease rather than an exclusively vascular disorder [7,8,32]. Beyond sustained glucose exposure, retinal injury arises from multiple interconnected pathogenic mechanisms, including oxidative stress, endothelial dysfunction, chronic low-grade inflammation, dysregulated lipid metabolism, and impaired tissue repair. Hyperglycemia triggers several key biochemical pathways, including activation of the polyol pathway, formation of advanced glycation end-products (AGEs), protein kinase C activation, mitochondrial dysfunction, and persistent inflammatory signaling. Recent evidence further highlights mitochondrial dysfunction as a key mediator linking oxidative stress, inflammatory activation, and retinal neurovascular injury, with dysregulation of mitochondrial stress-response pathways contributing to disease progression and representing a potential source of novel biomarkers [33]. Together, these processes contribute to the breakdown of the blood–retinal barrier, capillary non-perfusion, and progressive retinal ischemia. Rather than acting in isolation, these mechanisms interact synergistically, amplifying vascular injury, inflammation, oxidative stress, and neurovascular dysfunction, thereby creating a self-perpetuating cycle that drives the onset and progression of diabetic retinopathy.

### 4.1. The Role of Serum 25(OH)D

Serum 25(OH)D concentrations emerged as one of the strongest biomarkers associated with NPDR in the present study. Lower serum 25(OH)D concentrations were independently associated with higher odds of NPDR (OR = 0.9175 per 1 ng/mL increase; 95% CI: 0.8499–0.9904; *p* = 0.0273). The exploratory severity analysis further demonstrated differences in serum 25(OH)D concentrations according to retinopathy stage, with significantly lower levels in patients with moderate and advanced NPDR compared with patients without DR. However, a clear progressive decrease across all individual NPDR stages was not demonstrated, and the small number of patients with mild NPDR limits the statistical power of these subgroup comparisons. These findings therefore support a potential relationship between lower serum 25(OH)D concentrations and the severity of retinal microvascular disease, although they should be interpreted as exploratory.

Growing evidence suggests that vitamin D influences retinal homeostasis through several complementary mechanisms extending beyond calcium metabolism. Retinal endothelial cells, retinal pigment epithelial cells, Müller glia, and immune cells all express the vitamin D receptor, indicating that vitamin D signaling contributes directly to retinal physiology [34,35]. Although the mechanisms remain incompletely understood, available evidence supports a protective role of vitamin D in maintaining the blood–retinal barrier by reducing vascular permeability, leukocyte adhesion, and endothelial dysfunction [36,37]. Experimental studies have demonstrated that active vitamin D suppresses nuclear factor-kappa B (NF-κB) activation, decreases production of pro-inflammatory cytokines such as IL-6 and TNF-α, inhibits vascular endothelial growth factor (VEGF) expression, and preserves endothelial tight-junction integrity [36]. Moreover, vitamin D has immunomodulatory properties, promoting a shift from pro-inflammatory M1 macrophage polarization toward a more anti-inflammatory M2 phenotype while reducing activation of the NLRP3 inflammasome [38]. Vitamin D also exerts antioxidant effects by enhancing endogenous antioxidant enzyme activity while reducing reactive oxygen species generation [39]. Because oxidative stress is a central driver of retinal microvascular injury in diabetes, lower serum 25(OH)D concentrations may contribute to an environment in which oxidative and inflammatory damage initiated by chronic hyperglycemia is amplified. These immunomodulatory, antioxidant, and anti-inflammatory properties provide a plausible biological explanation for the inverse association observed between serum 25(OH)D concentrations and diabetic retinopathy.

Our findings are consistent with several observational studies and recent meta-analyses reporting lower serum vitamin D concentrations among patients with diabetic retinopathy, particularly those with more advanced disease [40,41]. Nevertheless, heterogeneity across published studies remains considerable, likely reflecting differences in ethnicity, geographic latitude, seasonal variation in vitamin D synthesis, dietary supplementation, renal function, assay methodology, and adjustment for diabetes duration and glycemic control. Consequently, although lower vitamin D status appears to be associated with increased retinopathy risk, whether supplementation can modify disease progression remains uncertain. Large randomized controlled trials are still required before vitamin D supplementation can be recommended specifically for the prevention or treatment of diabetic retinopathy.

The interpretation of categorical 25(OH)D status warrants consideration because thresholds for deficiency, insufficiency, and adequacy vary across clinical and research frameworks. In the present study, we used the 2023 updated clinical practice guideline definitions, classifying serum 25(OH)D concentrations < 20 ng/mL as deficient, 20–29 ng/mL as insufficient, and ≥30 ng/mL as sufficient [20,30], with concentrations < 12 ng/mL considered severely deficient [33]. In contrast, the D2d study used 50–74 nmol/L (20–29.9 ng/mL) as the adequate reference range according to the National Academy of Medicine and demonstrated that higher achieved 25(OH)D concentrations were associated with lower diabetes risk among participants receiving vitamin D supplementation [42]. These differences highlight the importance of interpreting categorical 25(OH)D classifications within the context of the guideline or analytical framework applied. In our study, the principal regression analyses treated serum 25(OH)D as a continuous variable, providing an assessment of its association with NPDR that was less dependent on categorical thresholds.

An additional consideration is that serum 25(OH)D concentrations may be influenced by lifestyle and behavioral factors that were not assessed in the present study. Patients with more advanced diabetic complications may have reduced outdoor activity or mobility and consequently less sunlight exposure, potentially contributing to lower endogenous vitamin D synthesis. Conversely, lower 25(OH)D concentrations may reflect metabolic or inflammatory processes associated with diabetes and its complications. Because information on sun exposure, outdoor activity, physical activity, dietary vitamin D intake, and supplementation was not systematically collected, the relative contribution of these factors cannot be determined. Moreover, given the cross-sectional design, reverse causation cannot be excluded. Therefore, the lower serum 25(OH)D concentrations observed in patients with NPDR should be interpreted as an association rather than evidence of a causal relationship.

BMI represents another potential determinant of serum 25(OH)D concentrations, as increased adiposity may be associated with lower circulating 25(OH)D through altered vitamin D distribution and metabolism [43]. However, BMI did not differ significantly between the NPDR and non-DR groups in our cohort, making a major BMI-related explanation for the observed difference less likely. Nevertheless, residual confounding by adiposity cannot be completely excluded, particularly because other lifestyle determinants of 25(OH)D status were not systematically assessed.

### 4.2. Systemic Inflammation in Diabetic Retinopathy

Chronic low-grade inflammation is increasingly recognized as a central contributor to the development and progression of diabetic retinopathy. In the present study, patients with NPDR exhibited significantly higher circulating IL-6 concentrations (*p* = 0.047), an increased NLR (*p* = 0.043), and a reduced LMR (*p* = 0.023), supporting the presence of systemic immune activation. These findings are consistent with the concept that chronic inflammation accompanies retinal microvascular injury and may contribute to disease progression.

Growing clinical evidence supports the utility of systemic inflammatory biomarkers as indicators of diabetic retinopathy. Composite hematological indices such as NLR and LMR integrate alterations in both innate and adaptive immune responses and have emerged as inexpensive, readily available markers of systemic inflammation in patients with T2DM [43,44,45]. Several cross-sectional studies and recent meta-analyses have demonstrated significantly higher NLR values in patients with diabetic retinopathy than in diabetic patients without retinal involvement, with progressively increasing values reported across more advanced stages of disease [43,44,45]. However, considerable heterogeneity exists regarding proposed diagnostic cut-off values, reflecting differences in ethnicity, age, diabetes duration, glycemic control, concomitant diabetic complications, and retinopathy severity. In a recent meta-analysis including 35 studies, Jin et al. [44] reported NLR thresholds ranging from 1.6–2.1 to 4.0–4.79, with higher values generally associated with proliferative disease.

Interestingly, platelet-derived inflammatory indices, including PLR and SII, were not significantly associated with NPDR in our study. Similar observations have been reported by Ji et al. [45], who found that although NLR increased with diabetic retinopathy severity, elevations in PLR and SII were primarily observed in patients with concomitant diabetic nephropathy, suggesting that these markers may reflect a greater systemic microvascular disease burden rather than isolated early retinal involvement. Our findings therefore suggest that leukocyte-related inflammatory pathways may be more readily detectable in early diabetic retinal involvement than platelet-related inflammatory responses. However, this interpretation should be considered hypothesis-generating, particularly because the present study was cross-sectional and the severity subgroup analysis did not demonstrate significant differences in NLR, PLR, or SII across NPDR stages.

Among the inflammatory biomarkers evaluated, IL-6 emerged as an independent factor associated with NPDR. Elevated circulating and vitreous IL-6 concentrations have consistently been reported in patients with diabetic retinopathy, with several studies demonstrating positive correlations with disease severity and the presence of diabetic macular edema [13,46,47,48,49]. IL-6 is produced by activated macrophages, endothelial cells, adipocytes, and Müller glial cells in response to metabolic stress and contributes to retinal inflammation by promoting endothelial activation, leukocyte recruitment, increased vascular permeability, and disruption of the blood–retinal barrier. Experimental evidence further suggests that IL-6 may contribute to retinal neovascularization through VEGF-independent mechanisms, highlighting its broader role in retinal vascular pathology. These observations have stimulated interest in IL-6 as a potential therapeutic target, and bispecific IL-6/VEGF monoclonal antibodies are currently being investigated for the treatment of diabetic macular edema, particularly in patients with incomplete response to standard anti-VEGF therapy [13,48,49].

In contrast, serum CRP concentrations were not significantly associated with NPDR in our cohort, despite previous reports demonstrating positive associations between CRP and diabetic retinopathy [17,50]. This discrepancy may reflect the nonspecific nature of CRP, whose circulating levels are influenced by obesity, metabolic syndrome, infections, and numerous other inflammatory conditions. Compared with CRP, IL-6 may more directly reflect inflammatory and endothelial activation pathways involved in retinal microvascular injury, which could explain its stronger association with NPDR in the present study.

Overall, our findings reinforce the evidence that systemic inflammation accompanies diabetic retinal disease and suggest that inflammatory biomarkers, particularly NLR and IL-6, may complement established clinical and metabolic parameters in identifying patients with NPDR. Nevertheless, these biomarkers should currently be regarded as supportive indicators of systemic inflammatory burden rather than independent decision-making tools. Prospective longitudinal studies are needed to determine whether changes in these markers predict disease progression and provide clinically meaningful information beyond conventional risk factors.

### 4.3. Lipid Profile, Atherogenic Indices, and Retinopathy Risk

The present study demonstrated that the LDL-C/HDL-C ratio was independently associated with NPDR (*p* = 0.03), whereas conventional lipid parameters, including total cholesterol, triglycerides, LDL-C, and HDL-C concentrations, did not differ significantly between patients with and without retinopathy. These findings suggest that the balance between atherogenic and anti-atherogenic lipoproteins may provide additional information regarding retinal microvascular involvement beyond isolated lipid measurements. Similar findings have been reported by Omran et al. [51] and Romero-Aroca [52].

Both study groups exhibited relatively elevated atherogenic index of plasma (AIP) values (0.456 ± 0.276 in the NPDR group and 0.473 ± 0.224 in the non-DR group), with no significant difference between groups. This finding suggests an overall unfavorable atherogenic profile in the study population but does not support a specific association between AIP and NPDR. Moreover, AIP was not retained in the final multivariable model.

One possible explanation for the association observed with the LDL-C/HDL-C ratio is that chronic hyperglycemia may affect the functional properties of circulating lipoproteins through glycation and oxidation, potentially impairing the antioxidant, anti-inflammatory, and endothelial-protective functions of HDL while promoting the vascular effects of modified LDL [53,54,55]. Such qualitative alterations in lipoprotein function could theoretically contribute to endothelial dysfunction, oxidative stress, and retinal microvascular injury despite relatively similar concentrations of individual lipid parameters. However, this interpretation remains hypothesis-generating because HDL functionality, oxidized LDL, ApoB, and cholesterol efflux capacity were not directly assessed in the present study. Further studies incorporating direct measures of lipoprotein quality and function are needed to determine whether qualitative lipoprotein dysfunction contributes to diabetic retinal microvascular injury.

### 4.4. Interplay Between Inflammation, Vitamin D Status, and Dyslipidemia

Beyond their individual associations with NPDR, serum 25(OH)D concentrations, systemic inflammation, and lipid abnormalities may represent interconnected components of a common pathophysiological pathway leading to retinal microvascular injury. Vitamin D has anti-inflammatory, antioxidant, and endothelial-protective effects, and lower vitamin D availability may favor activation of inflammatory signaling, including NF-κB and IL-6-related pathways. Conversely, persistent inflammatory activation may further promote oxidative stress, endothelial dysfunction, and disruption of the blood–retinal barrier. Dyslipidemia may amplify these processes through lipid oxidation, endothelial activation, leukocyte adhesion, and impaired vascular homeostasis. In particular, an unfavorable LDL-C/HDL-C ratio may reflect an imbalance between atherogenic and protective lipoprotein functions, increasing susceptibility of the retinal microvasculature to oxidative and inflammatory injury. These mechanisms may therefore interact rather than operate independently, with metabolic stress, inflammation, and impaired vascular protection reinforcing one another in the diabetic retina. The simultaneous independent associations of lower serum 25(OH)D concentrations, higher IL-6 levels, and a higher LDL-C/HDL-C ratio with NPDR in our cohort are consistent with this interconnected biological framework. However, these mechanisms remain biologically plausible rather than causally demonstrated by the present cross-sectional study.

### 4.5. Electrolytes and Metabolic Imbalances

No significant differences were observed between the NPDR and non-DR groups in serum calcium, magnesium, Ca/Mg ratio, iron, TIBC, TSAT, or sodium levels. Although the Ca/Mg ratio was elevated in both groups (>5), it did not differ significantly, suggesting that this imbalance may represent a characteristic of T2DM rather than a feature specific to NPDR. A recent study by Yücel et al. [55] found a positive correlation between a high Ca/Mg ratio and poor glycemic control, while Huang et al. demonstrated that an optimal Ca/Mg ratio of 2.0–2.5 is associated with reduced cardiovascular risk and recommended magnesium supplementation in older adults with T2DM [56,57].

Serum osmolarity was significantly higher in patients with NPDR. Because calculated serum osmolarity integrates glucose, urea, and electrolyte concentrations, it should be interpreted primarily as a composite marker of metabolic and renal homeostasis rather than as an independent biological pathway. The higher values observed in the NPDR group may therefore reflect the combined contribution of metabolic control, hydration status, and subtle renal alterations rather than an independent effect of osmolarity on retinal microvascular injury. Although additional adjustment for eGFR did not materially alter the principal associations observed in the multivariable model, the individual contributions of the components of calculated osmolarity cannot be completely separated in this cross-sectional analysis.

Recent epidemiological studies have reported associations between higher serum osmolality, inadequate hydration status, and diabetic retinopathy, suggesting that osmolarity may capture systemic conditions relevant to retinal microvascular health. In a study by Zhang et al. [25], including 5220 participants, a lower hydration status, defined by a serum osmolality ≥ 280 mOsm/kg, was associated with a higher prevalence of diabetic retinopathy. In our cohort, calculated serum osmolarity remained within the physiological range and was estimated rather than directly measured. Therefore, the modest increase observed in patients with NPDR may primarily reflect cumulative metabolic and renal disturbances rather than a distinct osmotic mechanism. Prospective studies are needed to determine whether serum osmolarity provides incremental information beyond its individual components and established metabolic and renal markers.

### 4.6. Strengths and Limitations

Several limitations should be considered when interpreting the findings of this study. First, the cross-sectional design precludes conclusions regarding causality or temporal relationships between biomarker alterations and the development of diabetic retinopathy. Second, the relatively small sample size and single-center setting may limit the generalizability of the results and increase the risk of selection bias. Although internal validation using 1000 bootstrap resamples provided supportive evidence of the stability of the model’s discrimination, the model was derived and internally validated within the same cohort and has not been externally validated. Consequently, the reported AUC should not be interpreted as evidence of established clinical predictive utility, and validation in larger independent cohorts remains necessary. The subgroup analysis according to NPDR severity should also be considered exploratory because the number of patients with mild NPDR was relatively small (*n* = 9), limiting the statistical power of comparisons between individual stages.

Several methodological considerations should also be acknowledged. Serum osmolarity was estimated using a validated formula rather than measured directly, and advanced biomarkers of lipoprotein function, including apolipoprotein B, oxidized LDL, HDL functionality, and cholesterol efflux capacity, were not available. Similarly, retinal inflammatory mediators and imaging biomarkers were not assessed, preventing direct correlation between systemic and local retinal inflammation. Potential residual confounding should also be considered. Although BMI was assessed and did not differ significantly between patients with and without NPDR, several other factors that may influence serum 25(OH)D concentrations, systemic inflammation, lipid metabolism, and serum osmolarity were not systematically collected, including the season of blood sampling, sunlight exposure, physical activity, dietary vitamin D intake or supplementation, statin use, previous anti-VEGF treatment, detailed diabetes medication classes, and systemic inflammatory conditions. Although patients with clinically diagnosed chronic kidney disease were excluded, renal function may still influence vitamin D metabolism and metabolic biomarkers. Additional adjustment for eGFR did not materially alter the principal associations observed in the multivariable model. Nevertheless, the absence of comprehensive information on these factors means that residual and unmeasured confounding cannot be excluded. Likewise, circulating IL-6 concentrations may be influenced by subclinical inflammatory conditions unrelated to diabetes. An additional methodological consideration is that diabetic retinopathy was classified at the patient level according to the more severely affected eye in patients with bilateral disease. Although this approach minimizes the risk of underestimating clinically relevant retinal involvement in asymmetric disease, it may result in a higher estimate of patient-level NPDR burden than single-eye classification strategies. Right-eye and left-eye sensitivity analyses were not performed because the study was designed primarily as a patient-level analysis, and the sample size was relatively small. Future studies with larger cohorts should evaluate the reproducibility of these associations using prespecified eye-level and patient-level classification strategies.

Finally, the relatively large number of biomarkers and clinical variables evaluated in relation to NPDR increases the possibility of type I error due to multiple comparisons. Because no formal multiplicity correction was applied, individual statistically significant associations should be considered exploratory and require confirmation in larger independent cohorts.

Despite these limitations, the study has several important strengths. To our knowledge, this is among the few studies to simultaneously evaluate nutritional, inflammatory, metabolic, electrolyte, and lipid-related biomarkers within a single cohort of patients with T2DM. The integration of routine biochemical markers with hematological inflammatory indices enabled a comprehensive assessment of multiple biological pathways implicated in diabetic retinopathy. Furthermore, the combined use of univariate analyses, receiver operating characteristic (ROC) analysis, and multivariable logistic regression provided a framework for evaluating the discriminatory and independent associations of the investigated variables. The consistent independent associations of lower serum 25(OH)D concentrations, elevated IL-6, and a higher LDL-C/HDL-C ratio with NPDR support the concept that diabetic retinopathy is a multifactorial disorder involving interconnected inflammatory, metabolic, and nutritional mechanisms.

An additional finding of interest was the strong association between the need for insulin therapy and NPDR. In our cohort, insulin requirement showed a stronger statistical association with NPDR than diabetes duration or HbA1c. Although insulin therapy cannot be considered a direct measure of pancreatic β-cell function, the need for insulin may represent a clinically accessible marker of greater treatment dependence and potentially reduced endogenous insulin reserve. In this respect, treatment intensity may capture aspects of diabetes severity that are not fully reflected by disease duration or HbA1c alone. Nevertheless, insulin use may also be influenced by treatment strategies, physician prescribing patterns, patient characteristics, and the clinical indication for insulin initiation. Therefore, residual confounding by indication cannot be completely excluded, and the observed association should not be interpreted as evidence of a direct relationship between insulin therapy, β-cell dysfunction, and retinopathy. Importantly, additional adjustment for eGFR did not materially change the associations observed for insulin therapy, serum 25(OH)D, IL-6, or the LDL-C/HDL-C ratio.

### 4.7. Clinical Implications and Future Perspectives

The present findings suggest that readily available biomarkers reflecting nutritional status, systemic inflammation, and metabolic dysfunction may complement established clinical risk factors for identifying patients with T2DM at increased risk of NPDR. Serum 25(OH)D, IL-6, and the LDL-C/HDL-C ratio remained independently associated with NPDR, while inflammatory indices such as NLR may provide additional information regarding systemic immune activation. Because these biomarkers are relatively accessible in clinical practice, they may contribute to more comprehensive risk stratification together with conventional predictors, including diabetes duration, glycemic control, blood pressure, and ophthalmologic assessment. However, they should be regarded as supportive risk-stratification markers rather than as independent decision-making instruments or substitutes for established ophthalmologic screening and clinical assessment.

From a therapeutic perspective, these findings support a comprehensive approach targeting established metabolic risk factors while recognizing potentially modifiable inflammatory and nutritional components. Optimization of lipid and glycemic control remains fundamental to reducing diabetic microvascular risk, whereas correction of low vitamin D status may be considered within general diabetes care. However, evidence is currently insufficient to recommend vitamin D supplementation specifically for the prevention or treatment of NPDR. Similarly, the association of IL-6 with NPDR provides a biological rationale for investigating anti-inflammatory strategies, but circulating IL-6 should currently be considered a marker of inflammatory activity rather than a therapeutic target in routine ophthalmic practice. Whether interventions targeting vitamin D status, lipid quality, or inflammatory pathways can modify the onset or progression of diabetic retinopathy requires confirmation in prospective interventional studies.

Future prospective multicenter studies involving larger and more diverse populations are required to validate these findings and determine the temporal relationship between systemic biomarker alterations and the onset or progression of diabetic retinopathy. Longitudinal investigations should evaluate whether serial measurements of serum 25(OH)D, IL-6, lipid ratios, inflammatory indices, and serum osmolarity improve the prediction of incident or progressive diabetic retinopathy beyond established clinical risk factors.

Future research should also investigate emerging biomarkers of lipoprotein quality and immune activation, including apolipoprotein B, apolipoprotein A-I, oxidized LDL, HDL functionality, paraoxonase-1 activity, and markers of inflammasome activation [58,59]. Integrating these biomarkers with retinal imaging modalities, such as optical coherence tomography angiography (OCTA), may improve understanding of the relationship between systemic metabolic disturbances and retinal microvascular injury.

Finally, the growing application of machine learning and multimarker prediction models offers an opportunity to integrate biochemical, inflammatory, metabolic, and imaging biomarkers into personalized risk prediction algorithms [60,61]. Recent advances in artificial intelligence further support the integration of multimodal clinical, laboratory, and retinal imaging data to improve individualized prediction and clinical decision-making in diabetic retinal disease [62]. Such approaches may facilitate earlier identification of patients at high risk of diabetic retinopathy and support individualized preventive strategies before irreversible retinal damage develops. Nevertheless, these models should undergo external validation and prospective clinical evaluation before being incorporated into routine clinical practice.

## 5. Conclusions

This study suggests that NPDR is associated with a combination of inflammatory, metabolic, nutritional, and osmotic disturbances rather than a single pathogenic mechanism. Lower serum 25(OH)D concentrations, elevated IL-6 levels, and a higher LDL-C/HDL-C ratio were independently associated with NPDR. The association between insulin therapy and NPDR likely reflects greater diabetes severity and treatment dependence rather than a direct effect of insulin therapy itself. These findings support the multifactorial nature of diabetic retinopathy and suggest that combining routinely available biomarkers may provide complementary information for identifying patients with NPDR. Prospective longitudinal studies and external validation in larger independent cohorts are needed to determine whether these biomarkers improve risk stratification beyond established clinical factors and whether multimarker models have potential clinical utility in routine clinical practice.

## Figures and Tables

**Table 1:** Baseline demographic, clinical, and biochemical characteristics of the study population.

Variable	NPDR (*n* = 55)	Non-DR (*n* = 43)	Total (*n* = 98)	*p*-Value
Age (years), mean ± SD	65.0 ± 8.9	68.3 ± 7.7	66.5 ± 8.5 years	0.06 ^1^
Females, *n* (%)	29 (52.7%)	27 (62.7%)	56 (57.1%)	0.48 ^2^
Duration of diabetes (years), mean ± SD	10.9 ± 6.5	9.3 ± 7.9	10.2 ± 7.1	0.079 ^3^
BMI (kg/m^2^), mean ± SD	25.3 ± 3.8	25.1 ± 4.1	25.1 ± 4.0	0.98 ^3^
Arterial hypertension, *n* (%)	40 (72.7%)	32 (74.4%)	72 (73.5%)	>0.99 ^3^
OADs, *n* (%)	28 (50.9%)	36 (83.7%)	64 (65.3%)	0.002 ^2^*
Insulin ± OADs, *n* (%)	34 (61.8%)	7 (16.2%)	41 (41.8%)	<0.001 ^2^*
FBG (mg/dL), mean ± SD	159.5 ± 70.7	128.4 ± 35.7	145.8 ± 59.5	0.079 ^1^
HbA1c (%), mean ± SD	7.6 ± 1.9	6.8 ± 1.2	7.3 ± 1.7	0.062 ^1^
BUN (mg/dL), mean ± SD	54.7 ± 34.3	43.6 ± 15.9	49.8 ± 28.1	0.406 ^1^
Creatinine (mg/dL), mean ± SD	1.2 ± 0.6	1.0 ± 0.3	1.1 ± 0.5	0.197 ^1^
eGFR (mL/min/1.73 m^2^), mean ± SD	73.2 ± 26.0	75.1 ± 21.0	74.7 ± 24.6	0.731 ^1^
eGFR < 60	18 (32.7%)	8 (18.6%)	26 (26.5%)	0.116 ^3^
Uric acid (mg/dL), mean ± SD	5.2 ± 1.6	5.9 ± 1.6	5.5 ± 1.6	0.031 ^1^*
ALT (U/L), mean ± SD	27.7 ± 20.1	22.2 ± 7.6	25.3 ± 16.0	0.883 ^1^
AST (U/L), mean ± SD	26.0 ± 14.9	23.7 ± 9.4	25.0 ± 12.7	0.838 ^1^

Footnote: 1—Mann–Whitney test; 2—chi-square test; 3—Fisher’s exact test; * statistically significant; OADs—oral antidiabetic drugs; FBG—fasting blood glucose; BUN—blood urea nitrogen; AST—aspartate aminotransferase; ALT—alanine aminotransferase.

**Table 2:** Hematologic and inflammatory indices.

Variable	NPDR (*n* = 55)	Non-DR (*n* = 43)	Total (*n* = 98)	*p*-Value
Neutrophils (×10^3^/µL, mean ± SD)	4.9 (±1.6)	4.8 (±1.1)	4.9 ± 1.4	0.92 ^1^
Lymphocytes (×10^3^/µL, mean ± SD)	2.1 ± 0.6	2.4 ± 1.0	2.3 ± 0.8	0.047 ^1^*
Monocytes (×10^3^/µL, mean ± SD)	0.9 ± 0.5	0.6 ± 0.2	0.8 ± 1.1	0.864 ^1^
Platelets (×10^3^/µL, mean ± SD)	231.9 ± 65.6	258.7 ± 71.6	243.6 ± 68.9	0.152 ^1^
NLR (mean ± SD)	2.8 (±1.2)	2.3 (±0.6)	2.6 ± 0.9	0.043 ^1^*
PLR (mean ± SD)	133.6 ± 55.8	123.2 ± 38.1	129.0 ± 48.6	0.596 ^1^
LMR (mean ± SD)	3.2 ± 1.2	3.7 ± 1.4	3.4 ± 1.3	0.023 ^2^*
SII (mean ± SD)	641.8 (±307.1)	589.6 (±250.4)	628.1 ± 280.6	0.33 ^1^
RBC (×10^6^/µL, mean ± SD)	4.5 ± 0.6	4.5 ± 0.4	4.5 ± 0.5	0.810 ^1^
RDW (%,mean ± SD)	14.2 ± 1.2	14.1 ± 1.0	14.1 ± 1.1	0.875 ^1^
Hemoglobin (g/dL, mean ± SD)	13.1 ± 1.6	13.0 ± 1.2	13.1 ± 1.5	0.781 ^2^
HRR (mean ± SD)	0.9 ± 0.2	0.9 ± 0.1	0.9 ± 0.1	0.997 ^3^
APTT (sec., mean ± SD)	29.0 ± 3.2	29.0 ± 3.3	29.0 ± 3.2	0.978 ^3^
PT (sec, mean ± SD)	11.4 (±1.7)	11.3 (±1.3)	11.4 ± 1.5	0.841 ^1^
INR (mean ± SD)	1.0 ± 0.2	1.0 ± 0.1	1.0 ± 0.1	0.566 ^1^
Fibrinogen > 4 g/L, *n* (%)	17 (30.9%)	11 (25.6%)	28 (28.6%)	0.723 ^4^
Fibrinogen (mg/dL, mean ± SD)	369.6 ± 76.3	343.1 ± 83.0	358.0 ± 79.6	0.078 ^1^
ESR (mm/h, mean ± SD)	18.2 ± 13.1	16.7 ± 14.0	17.6 ± 13.4	0.462 ^1^
CRP > 1 mg/L, *n* (%)	19 (34.6%)	18 (41.9%)	37 (37.8%)	0.595 ^4^
CRP (mg/dL, mean ± SD)	1.7 ± 3.1	1.4 ± 2.0	1.6 ± 2.7	0.437 ^1^
IL-6 (pg/mL, mean ± SD)	4.9 ± 3.1	2.8 ± 1.4	3.6 ± 2.6	0.047 ^1^

Footnote: 1—Mann–Whitney test; 2—Welch *t*-test; 3—Student’s *t*-test; 4—chi-square test; * statistically significant; NLR—neutrophil-to-lymphocyte ratio; PLR—platelet-to-lymphocyte ratio; LMR—lymphocyte-to-monocyte ratio; SII—systemic inflammation index; RBC—red blood cells; RDW—red cell distribution width; HRR—hemoglobin-to-red-cell-distribution-width ratio; APTT—activated partial thromboplastin time; PT—prothrombin time; INR—international normalized ratio; ESR—erythrocyte sedimentation rate; CRP—C-reactive protein; IL-6—interleukin-6.

**Table 3:** Vitamin D status, electrolytes, and metabolic indices.

Variable	NPDR (*n* = 55)	Non-DR (*n* = 43)	Total (*n* = 98)	*p*-Value
Serum 25(OH)D (ng/mL), mean ± SD	16.7 ± 7.5	21.0 ± 5.2	18.6 ± 6.9	<0.001 ^1^*
Serum 25(OH)D deficiency, *n* (%)	42 (76.4%)	19 (44.2%)	61 (62.2%)	0.002 ^2^*
Severe serum 25(OH)D deficiency, *n* (%)	16 (29.1%)	1 (2.3%)	17 (17.4%)	<0.001 ^3^*
Ca (mg/dL), mean ± SD	9.9 ± 3.1	9.6 ± 0.7	9.7 ± 2.1	0.228 ^1^
Mg (mg/dL), mean ± SD	1.8 ± 0.2	1.8 ± 0.2	1.9 ± 0.2	0.529 ^1^
Ca/Mg ratio, mean ± SD	5.5 ± 2.2	5.2 ± 0.8	5.3 ± 1.6	0.943 ^1^
Fe (ug/dL), mean ± SD	74.4 ± 35.1	72.7 ± 33.3	73.7 ± 34.0	0.892 ^1^
TIBC (ug/dL), mean ± SD	317.6 ± 58.6	331.5 ± 44.8	323.7 ± 52.9	0.146 ^1^
TSAT (%), mean ± SD	23.7 ± 11.5	22.5 ± 10.4	23.2 ± 10.9	0.861 ^1^
Na (mmol/L), mean ± SD	140.3 ± 4.3	140.4 ± 2.1	140.4 ± 3.5	0.381 ^1^
K (mmol/L), mean ± SD	4.6 ± 0.6	4.4 ± 0.4	4.6 ± 0.5	0.05 ^1^*
Osmolarity (mmol/L), mean ± SD	309 ± 14.2	303.4 ± 7.6	306.6 ± 12.1	0.033 ^1^*

Footnote: 1—Mann–Whitney test; 2—chi-square test; 3—Fisher’s exact test; *—statistically significant; Ca—calcium; Mg—magnesium; Na—sodium; K—potassium.

**Table 4:** Lipid profile and atherogenic indices.

Variable	NPDR (*n* = 55)	Non-DR (*n* = 43)	Total (*n* = 98)	*p*-Value
Total cholesterol (mg/dL), mean ± SD	166.6 ± 49.3	166.9 ± 42.8	166.8 ± 46.1	0.866 ^1^
Triglycerides (mg/dL), mean ± SD	145.9 ± 90.6	157.4 ± 70.6	150.9 ± 81.8	0.171 ^1^
HDL-C (mg/dL), mean ± SD	46.4 ± 14.3	49.4 ± 9.8	47.7 ± 12.5	0.070 ^1^
LDL-C (mg/dL), mean ± SD	98.4 ± 37.8	91.2 ± 41.3	95.2 ± 39.1	0.118 ^1^
Castelli index I, mean ± SD	3.8 ± 1.2	3.5 ± 0.9	3.6 ± 1.1	0.191 ^1^
LDL-C/HDL-C (Castelli index II), mean ± SD	2.3 ± 1.0	1.9 ± 0.9	2.1 ± 1.0	0.031 ^1^*
AIP, mean ± SD	0.5 ± 0.3	0.5 ± 0.2	0.5 ± 0.3	0.748 ^2^

Footnote: 1—Mann–Whitney test; 2—Student’s *t*-test; *—statistically significant; AIP—atherogenic index of plasma.

**Table 5:** Diagnostic performance of systemic metabolic and inflammatory biomarkers for NPDR in patients with T2DM.

Variable	AUC	SE	95% CI	Sensitivity	Specificity	Cut-Off Value
NLR	0.619	0.0575	0.515 to 0.715	47.3	83.7	>2.7
LMR	0.607	0.0578	0.504 to 0.705	38.2	91.4	<=2.6
Serum 25(OH)D (ng/mL)	0.712	0.0518	0.612 to 0.799	72.7	62.8	<19.2
IL-6 (pg/mL)	0.617	0.0570	0.514 to 0.714	72.7	53.5	>2.3
LDL-C/HDL-C ratio	0.627	0.0574	0.524 to 0.723	60	72.1	>1.9

Footnote: AUC—area under the curve; SE—standard error; CI—confidence interval; NLR—neutrophil-to-lymphocyte ratio; LMR—lymphocyte-to-monocyte ratio.

**Table 6:** Logistic regression model for NPDR.

Variable	Coefficient	Std. Error	Wald	OR	95% CI	*p*
Insulin therapy	1.50760	0.57185	6.9504	4.52	1.47 to 13.85	0.008 *
IL-6 (pg/mL)	0.40260	0.15805	6.4889	1.5	1.09 to 2.04	0.011 *
Serum 25(OH)D (ng/mL)	−0.086149	0.039035	4.8706	0.91	0.85 to 0.99	0.027 *
LDL-C/HDL-C	0.70173	0.27950	6.3035	2.02	1.17 to 3.49	0.012 *
Constant	−1.35197	1.13411	1.4211			0.233

Footnote: OR: odds ratio; CI: confidence interval; * statistically significant at *p*-value < 0.05.

**Table 7:** Sensitivity analyses of factors associated with NPDR after adjustment for diabetes duration and glycemic status.

Variable	Model 1: HbA1c-Adjusted		Model 2: FBG-Adjusted	
	OR (95% CI)	*p*	OR (95% CI)	*p*
Diabetes duration	0.995 (0.927–1.067)	0.879	0.997 (0.931–1.068)	0.928
HbA1c (%)	1.162 (0.831–1.623)	0.380	—	—
Fasting blood glucose	—	—	1.006 (0.997–1.016)	0.203
25(OH)D (ng/mL)	0.902 (0.834–0.976)	0.010	0.902 (0.834–0.974)	0.009
IL-6 (pg/mL)	1.384 (1.050–1.823)	0.021	1.383 (1.040–1.840)	0.026
LDL-C/HDL-C ratio	1.949 (1.150–3.304)	0.013	1.897 (1.111–3.240)	0.019

Footnote: OR: odds ratio; CI: confidence interval; FBG: fasting blood glucose; NPDR: non-proliferative diabetic retinopathy.

## Data Availability

The data presented in this study are available on request from the corresponding author.

## Supplementary Materials

The following supporting information can be downloaded at: https://www.mdpi.com/article/10.3390/biomedicines14081791/s1, Table S1: Correlation table for inflammatory biomarkers (SII, NLR < PLR, LMR, CRP, Il-6); Table S2: Correlation table for the biomarkers included in logistic regression model; Table S3: Subgroup analysis according to NPDR stage for the relevant biomarkers.
