# Supplementary material for: Association of Serum 25-Hydroxyvitamin D, Inflammatory, and Metabolic Biomarkers with Non-Proliferative Diabetic Retinopathy in Type 2 Diabetes Mellitus: A Cross-Sectional Study
Source: Biomedicines. 2026 Aug 9;14(8):1791. doi: 10.3390/biomedicines14081791 (PMC13509565; doi:10.3390/biomedicines14081791)
Supplement: Supplementary file 1 [file biomedicines-14-01791-s001.zip › biomedicines-4456772-supplementary.pdf]

Supplementary material 1

Table S1. Correlation table for inflammatory biomarkers (SII, NLR< PLR, LMR, CRP, Il-6)

SII	1	0.708 P<0.0001	0.703 P<0.0001	0.229 P=0.0271	0.027 P=0.7884	-0.313 P=0.0017
PLR	0.708 P<0.0001	1	0.488 P<0.0001	0.169 P=0.1048	0.063 P=0.5397	-0.395 P=0.0001
NLR	0.703 P<0.0001	0.488 P<0.0001	1	0.156 P=0.1345	0.109 P=0.2855	-0.607 P<0.0001
CRP(mg/dl)	0.229 P=0.0271	0.169 P=0.1048	0.156 P=0.1345	1	0.200 P=0.0549	-0.145 P=0.1666
IL-6(pg/ml)	0.027 P=0.7884	0.063 P=0.5397	0.109 P=0.2855	0.200 P=0.0549	1	-0.308 P=0.0020
LMR	-0.313 P=0.0017	-0.395 P=0.0001	-0.607 P<0.0001	-0.145 P=0.1666	-0.308 P=0.0020	1
	SII	PLR	NLR	CRP(mg/dl)	IL-6(pg/ml)	LMR

Spearman rank correlation coefficient

Footnote: NLR- Neutrophil-to-Lymphocyte ratio; PLR – Platelet-to-Lymphocyte ratio; LMR – Lymphocyte-to-Monocyte ratio; SII – Systemic Inflammation Index; CRP – C reactive protein; IL-6 - interleukine 6

Table S2: Correlation table for the biomarkers included in logistic regression model

LDL-C/HDL-C	1	-0.148 P=0.1449	0.122 P=0.2328	0.070 P=0.4937
IL-6(pg/mL)	-0.148 P=0.1449	1	-0.056 P=0.5808	-0.206 P=0.0420
insulin_therapy	0.122 P=0.2328	-0.056 P=0.5808	1	-0.297 P=0.0030
Serum 25(OH)D (ng/mL)	0.070 P=0.4937	-0.206 P=0.0420	-0.297 P=0.0030	1
	LDL-C/HDL-C	IL-6(pg/ml)	insulin_add_	Vit. D(ng/ml)

Spearman rank correlation coefficient

Footenote: Serum 25(OH)D – serum Vitamin D; LDL-C/HDL-C LDLper HDL cholesterol ratio;

Table S3. Subgroup analysis according to NPDR stage for the relevant biomarkers

	No DR<br>n = 43	Mild NPDR<br>n = 9	Moderate NPDR<br>n = 22	Severe NPDR<br>n = 24	P #
IL-6 (pg/mL)	2.3<br>2.0 to 3.4	3.7<br>2.0 to 6.3	3.7<br>2.6 to 4.6	2.9<br>2.5 to 3.8	0.1490
NLR	2.2<br>2.1 to 2.5	2.1<br>1.6 to 3.1	2.9<br>2.5 to 3.8	2.4<br>2.3 to 2.9	0.0532
PLR	122.3<br>104.2 to 131.9	132.2<br>96.3 to 207.0	121.4<br>107.0 to 192.0	124.0<br>99.2 to 135.2	0.7436
SII	577.9<br>491.9 to 649.9	566.8<br>371.8 to 855.8	653.8<br>525.7 to 798.5	623.3<br>473.0 to 742.7	0.6710
Serum 25(OH)D (ng/mL)	21.2[a,b]<br>18.6 to 22.2	17.8<br>8.5 to 24.5	18.1[a]<br>10.1 to 20.0	16.3[b]<br>15.0 to 18.0	0.0047
LDL-C/ HDL-C ratio	1.5<br>1.4 to 1.8	1.9<br>1.1 to 2.8	2.0<br>1.5 to 2.6	2.1<br>2.0 to 3.0	0.0742

Footnote: Data are: Median, 95% Confidence Interval; # Kruskal-Wallis test; [a] groups with the same letter differ significantly in the post-hoc test according to Dunn. NLR- Neutrophil-to-Lymphocyte ratio; PLR – Platelet-to-Lymphocyte ratio; SII – Systemic Inflammation Index; CRP – C reactive protein; IL-6 - interleukine 6; Serum 25(OH)D – serum Vitamin D;
